# Preliminary Study on the Diagnostic Performance of a Deep Learning System for Submandibular Gland Inflammation Using Ultrasonography Images

**DOI:** 10.3390/jcm10194508

**Published:** 2021-09-29

**Authors:** Yoshitaka Kise, Chiaki Kuwada, Yoshiko Ariji, Munetaka Naitoh, Eiichiro Ariji

**Affiliations:** Department of Oral and Maxillofacial Radiology, School of Dentistry, Aichi Gakuin University, Nagoya 464-8651, Japan; chiaki@dpc.agu.ac.jp (C.K.); yoshiko@dpc.agu.ac.jp (Y.A.); mune@dpc.agu.ac.jp (M.N.); ariji@dpc.agu.ac.jp (E.A.)

**Keywords:** deep learning, ultrasonography, submandibular gland, obstructive sialoadenitis, Sjögren’s syndrome

## Abstract

This study was performed to evaluate the diagnostic performance of deep learning systems using ultrasonography (USG) images of the submandibular glands (SMGs) in three different conditions: obstructive sialoadenitis, Sjögren’s syndrome (SjS), and normal glands. Fifty USG images with a confirmed diagnosis of obstructive sialoadenitis, 50 USG images with a confirmed diagnosis of SjS, and 50 USG images with no SMG abnormalities were included in the study. The training group comprised 40 obstructive sialoadenitis images, 40 SjS images, and 40 control images, and the test group comprised 10 obstructive sialoadenitis images, 10 SjS images, and 10 control images for deep learning analysis. The performance of the deep learning system was calculated and compared between two experienced radiologists. The sensitivity of the deep learning system in the obstructive sialoadenitis group, SjS group, and control group was 55.0%, 83.0%, and 73.0%, respectively, and the total accuracy was 70.3%. The sensitivity of the two radiologists was 64.0%, 72.0%, and 86.0%, respectively, and the total accuracy was 74.0%. This study revealed that the deep learning system was more sensitive than experienced radiologists in diagnosing SjS in USG images of two case groups and a group of healthy subjects in inflammation of SMGs.

## 1. Introduction

Among the various types of salivary gland lesions, inflammation is most common and has various underlying pathogeneses [1], including salivary flow obstruction, viral or bacterial infection, and autoimmune diseases such as Sjögren’s syndrome (SjS). Some of these diseases cause characteristic changes in the salivary gland parenchyma and are clearly visualized by ultrasonography (USG) [2,3,4,5,6,7,8,9]. Obstructive sialoadenitis is mainly due to sialoliths, most of which arise in the submandibular gland (SMG) or Wharton’s duct [2]. This condition can be diagnosed by USG, which exhibits a hyperechoic band and posterior shadow when a sialolith is present. However, the sialolith may not be detected in its early stage or when it is located adjacent to the inner surface of the mandible. Moreover, obstructive sialoadenitis occurs even in the absence of sialoliths. The parenchymal changes shown by USG in patients with this pathology vary depending on the disease stage. In the early stage, USG sometimes reveals no abnormality in the parenchyma, whereas other cases are characterized by enlargement and hypoechoic change. As the disease progresses, USG shows a mixed pattern with hyperechoic spots in the hypoechoic area, finally evolving to a homogeneous hyperechoic pattern that frequently exhibits atrophic parenchyma [7,8]. SjS is an autoimmune disease characterized by lymphocyte infiltration into exocrine glands such as the salivary glands and lacrimal glands, causing specific damage to these glands. USG findings are characterized by multiple small anechoic areas that frequently contain small hyperechoic spots within the inhomogeneous salivary parenchyma [7,8,9]. This appearance can be observed in both the SMG and parotid gland. These abnormal appearances of the SMGs may overlap depending on their stage among healthy individuals, patients with obstructive adenitis, and patients with SjS. In addition, the interpretation of images depends on the examiner’s experience level. Therefore, achieving a correct diagnosis is often difficult, especially for inexperienced observers.

Advanced deep learning systems have recently been developed, and their usefulness has been reported in the field of diagnostic imaging [10,11,12,13,14,15,16,17,18,19,20,21,22,23,24,25,26,27]. A deep learning system is a machine learning method used in artificial intelligence that allows a computer to learn tasks in the same way as humans. It is based on a neural network, which is a system that imitates the neurons in the human brain. We previously reported the diagnostic performance of a deep learning system for the differentiation of patients with and without SjS who complained of xerostomia using submandibular USG images [17]. Although our study revealed high diagnostic ability, inflamed SMGs can be present in individuals both with and without SjS, and this must be taken into account. Therefore, to ensure the effective use of deep learning systems for screening of individuals with a wide spectrum of clinical conditions, the performance of these systems should be investigated using individuals with definitively verified healthy, flow-obstructed, and SjS-affected glands.

The present study was performed to evaluate the diagnostic performance of deep learning systems using USG images that depict the parenchymal changes of SMGs affected by sialolith-induced obstructive sialoadenitis, SjS-affected SMGs, and normal SMGs.

## 2. Materials and Methods

The study design was approved by the Ethics Committee of Aichi Gakuin University, Japan (approval number 496) and was planned according to the ethical standards of the Helsinki Declaration.

### 2.1. Subjects

**Figure 1:**
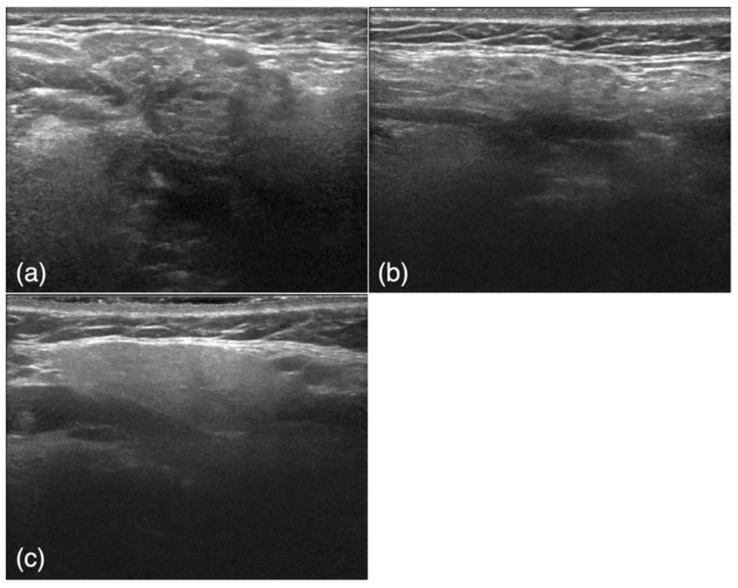
Examples of ultrasound images. (**a**) A patient in the obstructive sialoadenitis group shows inhomogeneous parenchyma and well-defined margins. (**b**) A patient in the Sjögren’s syndrome group shows inhomogeneous parenchyma characterized by multiple diffuse anechoic regions and ill-defined margins. (**c**) A patient in the control group shows homogeneous parenchyma and well-defined margins.

The subjects were retrospectively selected from an image database of patients who visited our institution from June 2010 to October 2019. The study included 50 USG images of 50 patients (26 men, 24 women; average age, 46.3 years) with SMG obstructive sialoadenitis confirmed by the presence of sialoliths using CT with inflammatory symptoms such as swelling and tenderness (obstructive sialoadenitis group) and 50 USG images of 28 patients (1 man, 27 women; average age, 66.0 years) with a confirmed diagnosis of SjS according to both the Japanese criteria [28] and the American–European Consensus Group criteria [29] (SjS group) (Figure 1a,b). The diagnosis of SjS was based on a blood test, sialography, and a Saxon test; in some cases, a biopsy was also performed if the above tests were inconclusive. Images of the affected side were obtained for patients with sialolithiasis, and images of both sides were obtained for patients with SjS. Among the obtained SjS images, six poor-quality images were excluded. USG scans were also obtained from 50 control subjects (26 men, 24 women; average age, 59.0 years) with no SMG abnormalities who had presented with other diseases, such as cervical lymph node metastasis of oral cancer or submandibular lymphadenitis (Figure 1c). The control subjects were randomly selected from the imaging database of our hospital retrospectively. The 150 images were randomly divided into training and test groups for the deep learning process. The training group comprised 40 images from the obstructive sialoadenitis group, 40 images from the SjS group, and 40 images from the control group. The test group comprised 10 images from the obstructive sialoadenitis group, 10 images from the SjS group, and 10 images from the control group.

### 2.2. USG Protocol

**Figure 2:**
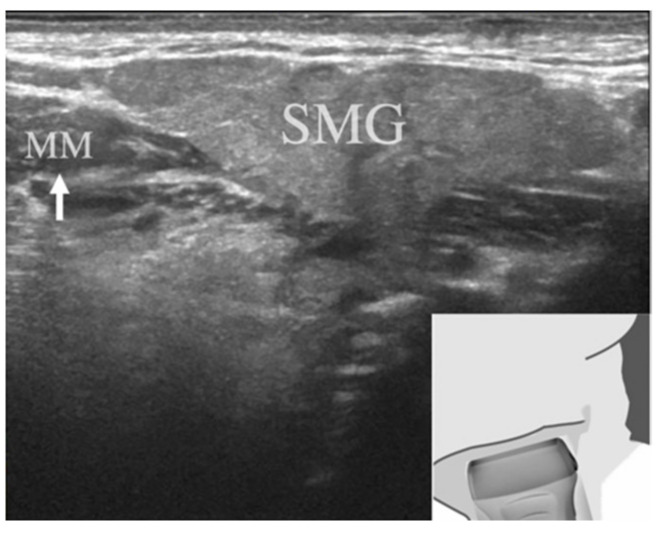
Illustration of right submandibular gland imaging. The submandibular gland was located in the center of the region of interest, and the mylohyoid muscle was located in the anterior-lower part of the submandibular gland (white arrow). MM, mylohyoid muscle; SMG, submandibular gland.

All USG images of the SMGs were taken in B-mode using a Logiq E9 system (GE Healthcare, Tokyo, Japan) with a center frequency of 12 MHz. The patient was placed in the supine position with his or her neck extended and face directed away from the examination site, and the scan was performed with the sagittal plane parallel to the inferior border of the mandible. The probe was oriented so that the SMG was located in the center of the region of interest and the mylohyoid muscle was located in the anterior-lower part of the SMG (Figure 2). Images of the entire SMG were saved as multiple still images in our hospital imaging database.

### 2.3. Imaging Data

USG images were downloaded from the hospital imaging database in Digital Imaging and Communications in Medicine (DICOM) format. In this study, we focused on parenchymal changes; therefore, the images in the obstructive sialoadenitis group were selected from among those without a sialolith in the region of interest. These USG images were selected by one radiologist (C.K.). The images in the SjS group were selected by one radiologist (Y.K.). A single radiologist (Y.K.) then converted the USG images from DICOM format to Portable Network Graphics (PNG) format. A five-fold cross-validation procedure was used to train the deep learning model for image classification [21,25]. The data were randomly split into five groups. One group was used as a testing set, and the residual data were used as training and validation samples. The validation samples were randomly assigned 25% from the training samples. We ensured that the training and testing data did not contain samples from the same image or the same patients while maintaining a balanced number of samples in each group. All datasets were created by one radiologist (Y.K).

We performed data augmentation on the training data [10,17,25]. Data augmentation is a frequently used technique for deep learning implementation with a small number of clinical cases and, in this technique, the number of data items is synthetically increased by altering the brightness, contrast, rotation, and sharpness of the images. In total, 2000 augmented images from the obstructive sialoadenitis group, 2000 augmented images from the SjS group, and 2000 augmented images from the control group were included in the analysis.

### 2.4. Diagnostic Performance of the Deep Learning System

The deep learning process was implemented on a NVIDIA GeForce GTX GPU workstation (Nvidia Corp., Santa Clara, CA, USA) with 11 GB of GPU and 128 GB of memory. The deep learning process was performed using VGG16 architecture that was pretrained using the ImageNet dataset for transfer learning. The VGG16 architecture contained 16 layers, which consisted of 13 convolutional layers and 3 fully connected layers. We created a model that collectively identified the obstructive sialoadenitis group, SjS group, and control group and performed the following procedure for a model. The training and validation processes were conducted for 30 epochs until sufficient learning rates were obtained. The automated selection method was repeated five times, resulting in five learning models. Each test data item was then input into each learning model, and whether each image represented a correct or incorrect response was determined with its probability. The probability was automatically calculated by the deep learning machine for each image. After this process, the results of the five learning models were summarized and the accuracy was calculated. We performed this process twice and summed the results to calculate the final accuracy.

To compare the diagnostic performance of the deep learning system, two experienced radiologists (Radiologists A and B), each of whom had more than 15 years of experience, evaluated the same images (50 obstructive sialoadenitis, 50 SjS, and 50 control images) after the calibration using 15 images (5 obstructive sialoadenitis, 5 SjS, and 5 control images) selected randomly from the training data sets before actual evaluations. For assessment of intraobserver agreement, the radiologists performed two evaluations at an interval of at least 1 month.

### 2.5. Statistical Analysis

Intraobserver and interobserver agreement was assessed with κ values using SPSS statistical software version 27 (IBM Corp., Armonk, NY, USA). A κ value of < 0.20 indicated poor agreement, 0.21 to 0.40 indicated fair agreement, 0.41 to 0.60 indicated moderate agreement, 0.61 to 0.80 indicated good agreement, and 0.81 to 1.00 indicated excellent agreement.

## 3. Results

Table 1 summarizes the results obtained by the deep learning system and those obtained by the radiologists. The sensitivity of the deep learning system in the obstructive sialoadenitis group, SjS group, and control group was 55.0%, 83.0%, and 73.0%, respectively, and the total accuracy was 70.3%. The sensitivity of the two radiologists in the obstructive sialoadenitis group, SjS group, and control group was 64.0%, 72.0%, and 86.0%, respectively, and the total accuracy was 74.0%.

The intraobserver agreement rates were good for both radiologists (κ = 0.64, 0.69) (Table 2). The interobserver agreement rates were moderate (first: κ = 0.60, second: κ = 0.53). The intermodel agreement rate was good (κ = 0.72).

## 4. Discussion

Salivary gland inflammation is caused by a variety of factors and is the most common of the major salivary gland lesions. USG is important for the diagnosis of salivary gland lesions, and its usefulness has been proven [3,4,5,6,7,8,9]. However, diagnosis via USG images is difficult, and its accuracy depends on experience. Many studies on imaging diagnosis using deep learning systems have been reported in recent years, proving that deep learning systems are effective in supporting image diagnostics. USG imaging has also been performed in various fields and is reportedly as accurate as radiologists’ assessments; however, only a few such reports have focused on the oral and maxillofacial area.

In this study, the accuracy of the deep learning system and that of the radiologists was almost identical, but it was not very high (about 70%). However, the sensitivity in each group was distinctive: the deep learning system showed higher sensitivity in the SjS group, the radiologists showed higher sensitivity in the control group, and both the deep learning system and radiologists showed low sensitivity in the obstructive sialoadenitis group. We previously evaluated the diagnostic performance of a deep learning system for the detection of SjS in USG images and found that the accuracy, sensitivity, and specificity of the deep learning system for the SMGs were 84.0%, 81.0%, and 87.0%, respectively [17]. The sensitivity of the radiologists in the present study was lower than that in the previous study. This presumably occurred because the present study involved three groups, including the obstructive sialoadenitis group. Therefore, USG images of the SMGs in patients with obstructive sialoadenitis and SjS may be similar depending on the stage of the disease, and the two conditions should be carefully distinguished from each other.

In the control group, the sensitivity of the radiologists tended to be higher than that of the deep learning system. Most radiologists start their training by first understanding normal images. Imaging diagnosis is then based on the normal image to determine whether an abnormality is present and to finally make a diagnosis. Therefore, an experienced radiologist is considered to be a specialist in normal images.

The sensitivity in the obstructive sialoadenitis group was low for both the deep learning system and radiologists and were the main reason for the low total accuracy. The USG findings of chronic sialadenitis vary with the stage of the disease: some show enlarged glands and dilated conduits, while others show multiple hypoechoic areas without enlarged glands. [3,8]. These findings suggest that obstructive sialoadenitis should always be included as a differential diagnosis because it presents with various aspects ranging from normal to abnormal images depending on its stage.

Regarding the difference in sensitivity between the deep learning system and the radiologists, the deep learning system learns from the images input to the neural network and then attains a diagnosis. The neural network is a black box, and its contents are therefore unknown; however, it might use some information different from that used by humans to make a diagnosis. The deep leaning system can be used as a second set of eyes for identification of such conditions, and in the future it may become more accurate with increased data-sets and better algorithms.

In the present study, the intraobserver agreement rates for both radiologists were good, while the interobserver agreement rate was moderate. The radiologists calibrated before evaluation, but if there were similar images, they are likely to set their own criteria and make decisions. As a result, it is considered that there was a gap between the observers and the interobserver agreement became worse. The agreement rate for the deep learning system was good and attained the highest value. Observer agreement rates are important for screening a wide range of subjects. The results of this study suggest that automatic diagnosis using a deep learning system may be useful. However, further improvement of the agreement rate is necessary, and this might be achieved by increasing the number of cases in future studies.

The present study had several limitations. First, we only used B-mode images and did not use sonoelastography images, Doppler images, or dynamic images. As sonoelastography would be potentially useful for the diagnosis of chronic inflammatory conditions of the major salivary glands as reported by others [4,5], its performance using a deep learning system should be verified in future studies together with Doppler mode sonography and dynamic images. Second, we did not include cysts or tumors in the evaluation of this study. Cysts and tumors often develop in the SMG, and USG is one of the most effective methods of examination. However, these lesions are easy to detect because they are not global changes in the gland parenchyma. Future studies will need to distinguish between cysts and tumors and between benign and malignant tumors. Finally, the number of patients was small. We compensated for this disadvantage by using data augmentation techniques and amplifying the images used for the training data; however, it is important to use abundant original data to improve the accuracy of the deep learning system. Given that there is a limit to the number of cases in one facility, collaboration with multiple facilities will be necessary.

## 5. Conclusions

This study revealed that the deep learning system was more sensitive than experienced radiologists in diagnosing SjS in USG images of two case groups with SMG inflammation and a group of healthy subjects. Further studies on deep learning systems with ultrasonography would be beneficial in improving the accuracy in identification of salivary gland inflammation.

## Figures and Tables

**Table 1:** Results obtained by deep learning system and radiologists.

		Obstructive Sialoadenitis	SjS	Control	PPV (%)
DL	Obstructive sialoadenitis	55	12	20	63.2
	SjS	29	83	7	69.7
	Control	16	5	73	77.7
	Sensitivity (%)	55.0	83.0	73.0	70.3 (accuracy)
Radiologists	Obstructive sialoadenitis	64	22	14	64.0
	SjS	26	72	1	72.7
	Control	10	6	86	84.3
	Sensitivity (%)	64.0	72.0	86.0	74.0 (accuracy)

DL, deep learning system; SjS, Sjögren’s syndrome; PPV, positive predictive value.

**Table 2:** Interobserver and intraobserver/model agreement.

Intraobserver Agreement		
Radiologist A	0.64	
Radiologist B	0.69	
Mean	0.66	Good
Interobserver/model agreement		
Radiologist A vs. B (first)	0.60	
Radiologist A vs. B (second)	0.53	
Mean	0.57	Moderate
DL (first) vs. DL (second)	0.72	Good

DL, deep learning system.

## Data Availability

The data presented in this study are available on request from the corresponding author. Names and exact data of the participants of the study may not be available owing to patient confidentiality and privacy policy.
